# Intelligence

**Published:** 1827-03

**Authors:** 


### INTELLIGENCE.

### MONTHLY REPORT OF PREVALENT DISEASES.

The cold bleak winds which have prevailed during the latter part of the last month, have given rise to abundance of pulmonary complaints, and Pneumonia and Pleurisy have been more frequent than at any former period of the season. In addition to these purely inflammatory cases, we have seen several instances in which continued fever has been combined with severe affection of the lungs, especially towards the advanced stage of the disease, by which the convalescence has been sometimes rendered extremely slow. The same state of atmosphere which has produced these symptoms, has likewise proved severe upon elderly persons labouring under "winter-cough." The habitual pulmonary affection has in them occasionally assumed a more acute form, requiring depletion, and in some cases proving fatal, by an overwhelming secretion of muco-purulent matter, from which the patients have been unable to clear the air-passages. In addition to these, we have met with several cases in which the larynx and trachea have been the seats of inflammation, both in children and in adults. We may therefore, with propriety, say, that inflammatory affections of the respiratory organs have been the prevailing diseases of the month.

*February 23d.*

### *Case of Obliteration of the Aorta, &c.*

Sir,—Some kind, but unknown friend has lately sent me several Numbers of a little work, published by the Medical Society of Tours. I therefore take the opportunity of acknowledging my obligations, by freely translating a case of Obliterated Aorta; and beg to remark, that the fact of that vessel being obliterated without producing death of the lower extremities, is the best answer that could be given to those who have abused Sir A. Cooper for his hitherto unexampled operation of tying it. I once tied the aorta of a dog with complete success; and, as nature performs her work most efficiently by enlarging anastomosing vessels, in a ratio with the increased necessity for their exertions, I cannot but hope that the day will ere long arrive when the same operation will be successfully performed on man. The case is interesting in other points of view; but the pathological descriptions are obscure, and difficult to comprehend. I am, Sir, your obedient servant,

JAMES M. CHURCHILL.

77, *Dart-street, Grosvenor-square.*

A woman named Cabaret, thirty-six years of age, and of a good constitution, had always enjoyed excellent health until six years ago, when she observed in the anterior and lower part of the arm, between the elbow and wrist, a moveable but not painful tumor, of the size of a hazel-nut, which gradually increased to that of an egg, and became the seat of violent shooting pains. After having in vain employed different means, the tumor was removed with the knife, by M. Dupuytren, in 1821, and healed very speedily; but returned soon after, and was then excised by M. Dubois. The healing of the wound seemed at first to proceed rapidly; but granulations occurring, recourse was had to arsenic paste, and the healing was accomplished in a few months. On the 24th of July, 1824, this woman felt some shooting pains a little above the old cicatrix, and soon after had some slight fits of coughing, attended with constriction in the chest, but without expectoration or any particular pain. The catamenia continued regular. She applied no remedy,—only took less food. In the month of October, the cough became violent, her breathing shorter; she was seized with paroxysms of suffocation; her appetite diminished, and sleep deserted her. She was ordered pectoral ptisans and opiates, but her sufferings increased. Towards the latter end of November, she was ordered saffron pills; and from that moment the symptoms acquired much intensity.

She was removed to the Hospital of St. Côme on the 9th of December. Her appearance differed little from a state of health; her tongue was white and moist; she had but little thirst, never any fever; her respiration was slow, short, and difficult. On percussion, her chest sounded much. The stethoscope *(le cylindre,)* showed that the air entered freely into the lungs. On the fore-arm there was a fixed tumor, unattended by pain, or by alteration in the skin. M. Bougon, the king's surgeon, thought there was cancer in the lungs, and prognosticated accordingly. The suffocating fits and the dry cough went on increasing, particularly towards night; the pulse remained calm; the abdomen was soft, and not tender.

On the 11th of January, 1825, the lower extremities appeared to be paralysed, and the patient died, though still somewhat corpulent.

The body was examined thirty hours after death. There were found—

1st. A schirrous swelling forming one mass within the radius, half an inch above the scar left by the second operation; tubercles of the same kind in the thyroid gland, and between the muscular fibres of the right thigh.

2d. Cancerous depositions in the tissue of the heart, and between the ribs and the pleura. In all points of both lungs were numberless hard round bodies, the largest not exceeding the size of a small hen's egg. Some appeared schirrous; others, in great number, had the character of a brain-like substance. Stony, calcareous masses occupied the mesentery: others, of a medullary nature, the omentum. Schirrous and cancerous tubercles were beneath the mucous membrane of the stomach, of the duodenum, of the small intestines, of the gall-bladder; likewise in the kidneys and the pancreas; while the liver appeared to be gorged with them.

3d. In the interior of the vena cava, a cylinder of a greenish colour, and of a fibrous texture; one of its branches having a cancerous appearance.

4th. From the third lumbar vertebræ, *the aorta was filled with a cylinder of a yellowish grey colour*, in which was observed a small quantity of puriform matter. *In the common and external iliacs, a substance offering most of the characters of old coagula.* The contexture of the vessels was not altered. *All the arteries proceeding from the aorta above the obliterated point were free, and of greater amplitude than usual.*

5th. Two tubercles, of the same kind with those of the other organs, on the internal surface of the dura mater. A third, of the size of a walnut, within the right occipital fossa, the pressure of which had produced absorption of the bone.

M. Velpeau, the senior clinical professor, considers this as one of the most interesting cases that can be stated, to show—1st, the alteration of the fluids in disease; 2dly, the possibility of obliterating the aorta, without producing the death of the lower limbs; 3dly, the diathesis or general disposition, known by the appellation "cancerous disposition;" 4thly, the origin of cancer from other than inflammatory causes.

---

### *Remarks on some of the Phenomena of Inflammation, as they appear on Dissection.*

For a considerable period, the fluids of the body were considered as the seat of diseases; at present they are believed to be seated in the solids or tissues. But can we now say, with all the additional light which has been of late thrown on diseases in general, that the blood, which performs so important functions, and which constitutes so large a portion of the body, is not affected by disease? and if so, have we any proofs of its not being primarily affected?

The opinions of authors regarding the supposed proximate cause of inflammation, seem to have been regulated by these two opinions. At one time it was supposed that it might depend on a congestion of blood or humours in the part, the large globules of blood plugging up the smaller vessels: whilst at present many believed that the proximate cause of inflammation may depend on a spasm of the extreme vessels, on a diminution of the muscular power of the arteries, or on an increase or diminution of the power of the capillary vessels of the part. Each of these opinions may be in part true, but it seems more probable to suppose that their authors either have imagined what might suit their own ideas, or have considered one of the primary effects of inflammation as its proximate cause.

It is the capillary vessels which are the seat of inflammatory diseases, but their great minuteness renders it impossible for the anatomist to examine them by the usual means, and the result of the observations made by the microscope are so often contradictory, that little reliance can be placed on their accuracy. It is not my intention to speculate on this very obscure, although most interesting subject; but, probably it may be allowed me to infer that the structure and functions of veins are nearly the same at different parts of their course, and that consequently the diseases of the different parts resemble each other.

By observing then the phenomena of inflammation in the large veins, it appears to me that some conclusions may be drawn as to the diseased change in the extreme vessels. When a vein has been wounded, as in bleeding, it sometimes happens that it inflames, the wound does not heal, and a discharge of a purulent fluid takes place from the orifice. This I have seen happen twelve hours after the operation of phlebotomy; and, although a considerable wound remained open in the vein, it did not bleed. The inflammation extended, and the patient was destroyed without his having had any hemorrhage from the wounded vein. This absence of hemorrhage might be supposed to be owing to the adhesion of the opposite sides of the vein; but, on examination after death, it was found to be produced by the vein being plugged up by consolidated* blood, some distance above and below the wounded part. In the same manner may be explained the reason why hemorrhage does not take place when a part is destroyed by mortification,—from the inflammation which preceded this state producing a consolidation of blood in the vessels. A few days ago I had an opportunity of seeing an interesting example of this kind at St. Bartholomew's Hospital, in which the extreme parts of the body had mortified without any assignable cause. When the body was examined after death, it was found that the principal veins of these parts had their vasa vasorum enlarged, their coats thickened, and the internal surface of a deep red colour, which was firmly attached to a consolidated portion of blood, which completely obstructed the vessels.

Such a consolidation of blood appears to be a constant effect of a certain degree of inflammation in the branches of veins; and, as the structure and contents of these vessels seem to be the same, may I not be allowed to infer that the same cause will produce the same effect, and that, therefore, a like consolidation of blood takes place in the capillary veins? This is not a mere speculation, as various proofs might be brought forward in its support. The cause of inflammation acting on the capillary vessel produces this consolidation of blood which impedes the circulation in the vessel; and, as the effect increases in extent, other changes in the part are the consequence. The arterial vessels continue to secrete, whilst the veins do not absorb in the same proportion, and a swelling is the consequence: the arteries meeting with an impediment in the part, propel their contents with greater force; whilst the smaller branches enlarge, to carry on this imperfect circulation, and produce the redness of the part.

It is on account of these changes that the power of absorption is diminished in inflamed parts, and that they are injected with much greater difficulty than in the healthy state.

It is the absence of this consolidation of blood which constitutes the difference between parts which may exhibit some of the phenomena, but not all, of inflammation, and parts which are actually inflamed: thus we explain why the determination of blood to the uterine system during pregnancy, or to the stags' horns during their growth, is not a state of inflammation. In the same manner, the influence of the mind on particular parts, as on the face in blushing, or on the erectile tissue, producing a determination of blood and other characteristic phenomena of inflammation, without the part being in that state, as the blood is not consolidated in the vessels.

THOMAS A. WISE.

*St. Bartholomew's Hospital; February* 10, 1827.

---

### *Salivation speedily produced by Inhaling Mercurial Vapour.*

Sir,—If you think the following circumstance worthy of a place in your interesting Journal, you may insert it. I would only premise, that the correspondent, Captain Sykes, of the Indian army, is an accurate and faithful observer. In a letter which I have lately received from him, dated at Poona, on the 28th of August, 1826, he says—

"An accident occurred in my laboratory on the 6th of August, which has led to the knowledge of a fact with respect to the native practice of medicine, which I believe to be new. I had occasion to distil some very impure mercury, for the purpose of filling my barometers. Some lime was put into the iron retort, together with the mercury; the receiver was properly luted on, and a charcoal fire applied. Being suddenly called away, I did not hesitate to leave the apparatus under the charge of a Hindoostanee moonshee, who pretends to knowledge of alchemy, he having succeeded perfectly in a

* I employ this word in opposition to the term coagulation, which is generally employed for designating the chemical changes which the blood undergoes when removed from the system.

similar distillation a few days before. On my return to the laboratory in about two hours and a half, I found every thing in it, and in the office (an adjoining apartment), as well as the people in both places, covered over with a bluish grey powder, which, on being swept together, formed globules of mercury. There were six persons in the two rooms: the moonshee, and a native who supplied the furnace, were in the laboratory; and four persons were in the office, two seated on chairs, and two on carpets on the floor. In the partition-wall between the two apartments, there is an open door, and two open windows. I found the moonshee and the English writer complaining of tenderness and pain under the jaws, and soreness in the joints. The English writer had been seated on a chair, above the level of the retort, in the office; and the moonshee had been standing near the furnace. The man who supplied the fire did not complain at the time, but in three hours he was reported to be ill, and unable to move. The other persons had no complaint at the time; but next morning, at ten o'clock, the whole six were suffering from sore mouths, and the severity of the symptoms seemed to be in the ratio of their distance from the retort at the time of its explosion, and of the elevation of their heads above its level. He who supplied the fire had his head and fauces so much swollen, that he could not speak; the moonshee and English writer were suffering severely; and a European writer, whose system had resisted forty grains of calomel a-day, had been spitting all night. The two who had sat below the level of the retort suffered much less than the others. My friend, Dr. Ducat, satisfied himself that they were cases of mercurial salivation, and of very unusual severity particularly in the person who had supplied the fire.

"It appeared that, in the progress of the distillation, some lime had got into the tube of the retort, and thus prevented the passage of the mercurial vapour, which had forced its way by the side of one of the screws by which the tube was fixed to the retort, and, diffusing itself through the air of the apartments, had been inspired by the men.

"The retort originally contained about six pounds of mercury, whereof half had escaped in the form of vapour.

"This is the twenty-second day since the accident occurred, and all the people are well, with the exception of the man who supplied the furnace.

"Our medical men produce salivation in twenty-fours, by fumigation; and I have heard of a recent instance of salivation being produced in seven hours, by fumigation.

"My Shastree, a learned Bramin, asserts, that the practice of producing salivation by means of mercurial vapour inhaled by the lungs has been used by the Hindoos from time immemorial. Bees'-wax is melted, and spread over stripes of thin cotton cloth; an equal quantity of cinnabar, in the form of powder, is spread over the waxed stripes of cloth, which are then rolled up in the shape of candles. The person to be salivated is seated on his haunches on the ground; a blanket is thrown over him; the lighted cinnabar candle is placed under the blanket, so that he inhales the mercurial vapour. A finger's breadth of the candle is burnt to salivate a boy; three or four fingers are required for a lad, and six for a robust man.

"When I expressed some doubt of this practice being of such antiquity among the Hindoos, my Shastree pointed out a passage in the 'Shaarungu Dhur,' an ancient Shanscreet work, in which the process is described."

I remain yours truly, W. SOMERVILLE.

*22, Princes Street, Hanover Square.*

---

### *Practical Remarks on the Utility of Fumigations.*

Upwards of three years' exclusive practice with this remedy enables me to lay before the profession some of the results derived from this experience. Concise as these observations may be, I beg to state they are such as may be relied on by those of the profession who have not yet given much of their attention to the subject: to those, on the contrary, who have been in the habit of resorting to this remedy, the subsequent statements will probably contain nothing new.

Many of the profession, either from cautious scepticism or occupation, have neither leisure nor inclination to enter into the details of the process, or of its merits. This may be readily excused when it is considered in what an empirical way, and with what vain pretensions the remedy was first introduced in this country; but such persons cannot have read the conclusive evidence on this subject, by the most distinguished of the faculty and official authorities of the neighbouring continent.*

In these observations, I shall not be deterred from stating in what diseases, or in what stages of disease, my experience with this remedy has been, and continues to be, at variance with the official reports of those authorities. In doing this, I feel I shall not detract from the merits of the remedy; for, if it is curative of only one disease, that is sufficient to establish it on a firm basis. To the encomiums bestowed on this remedy, I add my humble testimony, as far as the treatment is confined to most diseases of a chronic nature; those of the simpler kind, the fumigations are frequently sufficient to cure without the aid of medicine: but, on the contrary, for those of the acute type this remedy is altogether inadmissible, without the conjunct aid of the customary appropriate medicines. This will show how necessary it is to discriminate the cases submitted to this mode of cure; for the rationale of which I must refer the readers to former communications of mine on the subject, and to the authors before quoted. Without judicious selection in the cases submitted to this remedy, though so valuable a one, it may be the source of the greatest mischief.

In the under-mentioned chronic diseases of the skin, I have not found the remedy, as usually administered, to be so decidedly efficacious as stated by the authorities referred to.

In Prurigo Senilis, and other pruriginous complaints, the fumigating bath, at the usual temperature, (from 110 to 120 Fahr.) I have conceived to be a source of too much excitement, frequently producing aggravation of the complaint; but when the patient is submitted to what I may call a cold fumigation, (say as low a temperature as will disengage the sulphurous gas,) the troublesome itching quickly yields.

In Psora, (which term I would confine to the vesicular and papular stages of itch,) I believe this remedy possesses no advantages over the customary modes of cure; save that by this mode may be avoided all the unpleasantness of confinement, dressing with ointments, &c. I have generally found seven baths the average number required to effect the cure; and, the quicker they are taken in succession, the more speedily will this take place.

In Scabies Purulenta, in the early stages, when the pustules have a raised, inflamed, hardened base, this remedy does little good, unless assisted by frequent purges. In the latter or mixed stages of this complaint, Scabies Cachectica, the remedy is decidedly more useful. Medicines had likewise better be conjoined; and even then the complaint is liable to return in the spring and autumnal seasons.

In all the dry, rough, and scaly diseases, this remedy is comparatively beneficial in the order in which they follow—Ephelis, Pityriasis, Icthyosis: these complaints yield with the greatest facility, though of ever so long standing. In Psoriasis, particularly of old date, this remedy is equally serviceable; but, when resorted to in the early stages, as one part gets well, there are fre-

* See Memoirs and Reports on the Efficacy of Sulphurous Fumigations, from the French; published by order of that Government, and translated by Rees Price. 1818.

Observations on Sulphurous Fumigations, by William Wallace, Professor of Anatomy and Surgery. Dublin, 1820.

Researches on Chlorine Fumigations, by the same Author. 1822.

The Utility and Importance of Fumigations considered, by the Writer of this Paper. 1826.

quently fresh attacks of the disease in other parts, which however progressively get well.'

In the various degrees of Lepra, from Psoriasis to Elephantiasis, I believe this to be the best auxiliary remedy that can be resorted to; but, with all the aid of therapeutic agency, every practitioner knows the obstinate nature of these diseases. The spots and patches of these disorders, at first, I generally find extend under the use of this remedy; then become pale in the middle, gradually forming a large ring, which becomes broken through in parts before they disappear. I find this process facilitated by occasionally touching the edges with a pencil moistened with dilute acid.

In the Moist or Impetigonous Complaints of the Head, this remedy shows its advantages in a very short time. A lady, (a patient of Dr. GORDON and Mr. BRODIE,) seventy-four years of age, was covered from the head to the insteps as if she had had a blister over the whole of the body. The disease had existed eighteen months; various judicious courses of medicine had been unavailing. She commenced this remedy, and in three weeks became well. She took the fumigations in quick succession, one daily, with but little medicine during the trial, save occasionally an aperient.

In Impetigo, this remedy is eminently successful, but it is surprising to observe the various characters the disease sometimes assumes before it yields. I have had patients covered with impetigo, and before the ichorous discharge had quite subsided, ringworm in various parts has appeared,—in other parts pustules,—and again vesicles, varieties of herpetic appearances, and intertrigo, have followed each other, not observing any regularity; and frequently all these appearances have been distinct on the skin at the same time. The vesicles will come and disappear in a few hours; and the pustules are seldom painful; they maturate and die generally in twenty-four or forty-eight hours, if the fumigations are continued: indeed, in the progress of amendment in this complaint, I have seen the skin put on almost all the varieties of cutaneous disease,—each successively giving way to this remedy; for with these patients medicines have usually been given up as of little service. On the contrary, for diseases of the order Exanthemata, or eruptive diseases attended with fever, this remedy is inadmissible without the conjoint aid of medicine; and even then, as such diseases are generally of short duration, and curable by the usual means, I think this remedy may be as well omitted.

In Ecthema, where the inflammatory appearances are superficial and partial, showing as it were the want of power in nature to throw off the offending cause, and in those diseases consequent on Syphilis, or arising perhaps from the abuse of mercury, this is a remedy strongly to be advised. A weak state of constitution should not prevent its trial or adoption, for the judicious application of the remedy is decidedly tonic. *In short, I would say, for chronic cutaneous disease, this is the best remedy that can be resorted to; and in all diseases of the skin it is an auxiliary deserving consideration, as it does not prevent the administration of the customary modes of cure, but, from the temporary impulse that is occasioned to the whole of the animal functions, medicines are rendered more effectual in their operation.*

The generally received opinion, that this remedy is indicated for skin diseases only is to be regretted. It is for chronic diseases generally, and those of an anomalous nature, that this remedy ought to be valued, and, as a safe and useful auxiliary, should not be lost sight of. I do not hesitate to affirm, that this remedy is, comparatively speaking, less serviceable for skin complaints than for those I have just named.

Fumigations, it should be recollected, are not confined to the gas from sulphur only: chlorine and mercurial gases, and the vapour from narcotic or aromatic vegetables, can with equal facility be administered. The process consists in the patient's being seated in an apparatus, which is kept at about 98 or 100° Fahr., so that the patient shall not feel cold on entering the box. After he has been thus seated four or five minutes, the capillary vessels of the skin are forced into increased action, the pulse quickens, becomes full, though always soft, as the blood is equally distributed, and made to circulate in the most minute vessels on the surface; the face becomes flushed; and, during this quickened action of the bodily functions, the gas or vapour ascends from beneath the patient, surrounding the whole of the body,—the face only being excluded. The patient remains in the box about twenty minutes: during the last eight or ten he is under the full excitement of the bath; the absorbent system, simultaneously with the exhalants, are in increased activity; and, agreeably with physiological conclusions, the medicines are absorbed during this state of excitement; but more particularly is this the case when there is abrasion on the surface of the skin.

This excitement, if continued too long, would be mischievous; but the temporary acceleration that is thus given to the circulating fluids is found to be a mean of treating with success many simple diseases, and a process by which the powers of medicine are much aided in complaints of a more obstinate nature,—as in obstructions of the glandular system, torpor or inactivity of the liver, the varieties of rheumatism, atonic gout, headaches, skin complaints, &c.

The benefit that is to be derived from the use of the simple warm air bath, likewise deserves to be considered. When I first began to pay attention to this subject, I soon became impressed with the greater degree of exhilaration and comfort that usually follow the use of the dry bath, compared with the feelings after the use of the vapour bath, which are frequently those of languor. To this difference my attention was afterwards more particularly directed by the late Mr. Pearson, of Golden-square, who was always in the habit of directing the dry bath for those cases where debility was a prominent feature, or where there was languid circulation and deficient secretions. I have many times had reason to be satisfied with the preference given to these dry baths, under circumstances in which I am certain the vapour baths would not have been so beneficial. Mr. Pearson conceived that the moisture in the vapour bath, covering the surface of the body, might prevent the free action of the exhalant and other capillary vessels of the skin.

I shall conclude these observations by saying, that there are very few diseases for which these baths may not be found an useful auxiliary, except in organic diseases of the heart and its contiguous vessels, and in visceral inflammation. The temporary impulse given to the circulation augments all the secretions, and by so doing gives essential aid to the powers of medicine. I have had two cases of tertian ague, each of which had existed for more than a year, in which these baths proved successful. The patients were placed in the simple hot air bath on the commencement of the paroxysm, which remedy was, in both instances, completely effectual in preventing a recurrence.

J. GREEN.

*Great Marlborough Street; February.*

---

### *Case in which Constitutional Effects arose from the external Application of Belladonna.*

The following letter is from Mr. Wade, Apothecary to the Westminster Dispensary:—

Sir,—Much has of late been said in favour of the Extract of Belladonna as an external application, and, from its unquestionably, in many cases, powerful effect in diminishing irritability and excessive vascular action, it has a fair chance (considering the attention it has lately excited) of becoming a fashionable remedy in superficial inflammations. At present its powers are apparently much over-rated; for, as is the case with every thing in medicine which has even a show of novelty to recommend it, many virtues are attributed to it, and its faults, if seen, are overlooked, and considered as the result of our own mismanagement. The remedy will soon, however, find its proper level; and, in the hope of preventing its indiscriminate use, I am induced to relate the following case, which will be sufficient to show that, when used to abraded surfaces, the effect of the extract requires vigilant attention.

A gentleman consulted me lately, who had been annoyed for a long time with an obstinate cutaneous affection (Psoriasis) on the fore part of his wrist; the patch was about two inches and a half in length and breadth. Having learnt that almost every means of relief, both constitutional and local, had been tried with but little benefit, and having seen the Belladonna of use in similar cases, in which no absorption was evident, I was induced to recommend its trial in this. A plaster made with one part of the extract to two of soap-cerate was applied to the sore. About thirty-eight hours after its application, I was sent for, during the night, to this patient; whose countenance, on my arrival, expressed much alarm; his pulse was small and quick. He informed me that he had felt very languid during the previous day, and had taken his dinner without appetite; in the evening, he was much distressed by extreme distention of the stomach and bowels, giddiness and weight in the head, with dimness of sight. To relieve the distention of stomach, he took a glass of brandy and water, which produced great excitement, with extreme restlessness, which increased after his retiring to bed: he had constant nausea and retching, with a burning sensation in the throat. I found him sitting up in bed, making strong and ineffectual efforts to vomit: the pulse was small and frequent; the eyes had a dull, heavy expression; the pupils were much dilated, and had little action. The mucous membrane, from the transverse palatine suture, extending down the throat as far as could be seen, was of a deep purple hue. He complained of much giddiness, with an appearance of mist before his eyes; and said that, when attempting to get out of bed, his legs had failed him.

As the sense of distention was very distressing, and supposing that scarcely any digestion of the food last taken could have occurred, the muscular power of the stomach being apparently nearly lost, as nothing but a little mucus had been thrown up, I desired a strong emetic to be given to him, followed by full doses of ammonia. A considerable quantity of undigested food was soon vomited, which afforded much relief.

The restlessness, and burning sensation in the throat, continued more or less during the night. In the morning, but little uneasiness of stomach remained; the mucous membrane of the throat was of a dark crimson colour; the tonsils were much enlarged; the heat had, however, much diminished; the pulse was now sixty-five, full and soft; the pupils continued dilated. The patient complained chiefly of languor and a slight dimness of sight. The bowels had acted freely.—The ammonia was continued, and a capsicum gargle used to the throat.

It is useless to occupy more time in describing the symptoms or treatment, as all the ill effects of the Belladonna had in three days completely subsided.

A doubt might have existed in this case, whether the symptoms were really caused by the Belladonna, had not the patient himself settled this point beyond all question by again applying the plaster; being very anxious to get rid of his disease, and thinking that the symptoms might have arisen merely from a disordered stomach. It had not been applied many hours before the giddiness, dimness of sight, and langour, in some degree returned; but which quickly disappeared on the removal of the plaster.

*January* 16*th*, 1827. R. WADE.

### METEOROLOGICAL JOURNAL,

*From January 20th, to February 20th,* 1827.

By Messrs. Harris and Co. Mathematical Instrument Makers, 50, High Holborn.

| January | Moon. | Rain gauge. | Thermom. | | | Barometer. | | De Luc's Hygrom. | | Winds. | | Atmospheric Variations. | | |
|---|---|---|---|---|---|---|---|---|---|---|---|---|---|---|
| | | | 9 A.M. | MAX. | MIN. | 9 A.M. | 10P.M. | 9 A.M. | 10P.M. | 9 A.M. | 10PM. | 9 *a.m.* | 2 *p.m.* | 10 *p.m.* |
| 20 | | | 28 | 34 | 25 | 30.08 | 29.90 | 96 | 86 | E | E | Fair | Fair | Snow |
| 21 | ☾ | | 27 | 28 | 26 | 29.72 | 29.57 | 84 | 95 | NE | N | Snow | Snow | —— |
| 22 | | | 29 | 29 | 21 | 29.45 | 29.52 | 95 | 85 | E | NNE | —— | —— | Fine |
| 23 | | | 28 | 30 | 28 | 29.52 | 29.41 | 90 | 93 | NNW | W | —— | Fair | Cloudy |
| 24 | | | 30 | 33 | 28 | 29.46 | 29.52 | 90 | 93 | WSW | SW | Cloudy | —— | —— |
| 25 | | | 31 | 31 | 18 | 29.52 | 29.58 | 93 | 93 | E | ENE | Foggy | —— | —— |
| 26 | | | 28 | 33 | 30 | 29.58 | 29.68 | 93 | 88 | W | N | —— | —— | Some S. |
| 27 | ● | | 33 | 34 | 25 | 30.03 | 30.15 | 81 | 89 | NNE | NNE | Fair | —— | Fair |
| 28 | | | 33 | 42 | 42 | 30.05 | 29.84 | 87 | 96 | SW | SW | —— | —— | —— |
| 29 | | | 44 | 46 | 35 | 29.79 | 29.75 | 98 | 90 | SW | W | Cloudy | —— | —— |
| 30 | | | 39 | 41 | 39 | 29.67 | 29.63 | 90 | 88 | SSW | SW | —— | —— | Cloudy |
| 31 | | | 42 | 46 | 42 | 29.61 | 29.62 | 98 | 98 | SSW | SSW | Sm. Ra. | Cloudy | —— |
| Feb. | | | | | | | | | | | | | | |
| 1 | | | 43 | 44 | 35 | 29.64 | 29.67 | 96 | 96 | SSW | NNE | Cloudy | —— | Sm. Ra. |
| 2 | | | 36 | 42 | 29 | 29.81 | 29.96 | 81 | 85 | ENE | N | Fair | —— | Fine |
| 3 | ☽ | | 32 | 34 | 27 | 30.30 | 30.40 | 75 | 79 | NE | NE | —— | Fair | —— |
| 4 | | | 36 | 38 | 34 | 30.41 | 30.40 | 79 | 81 | NE v. | NNE | —— | —— | —— |
| 5 | | | 36 | 38 | 28 | 30.36 | 30.27 | 83 | 84 | ENE | ENE | —— | —— | Fair |
| 6 | | | 33 | 41 | 34 | 30.16 | 30.21 | 93 | 90 | NNE | NNE | Sm. Ra. | —— | —— |
| 7 | | | 37 | 41 | 30 | 30.29 | 30.31 | 90 | 85 | NE | NNE | Fair | —— | Fine |
| 8 | | | 35 | 37 | 29 | 30.34 | 30.35 | 78 | 80 | E | NE | —— | —— | —— |
| 9 | | | 32 | 36 | 28 | 30.24 | 30.18 | 77 | 75 | ENE | NE | —— | —— | —— |
| 10 | | | 34 | 37 | 30 | 29.97 | 29.93 | 82 | 87 | NE | NE | —— | —— | Cloudy |
| 11 | ○ | | 31 | 33 | 31 | 29.72 | 29.69 | 86 | 86 | ENE | ENE | Cloudy | Cloudy | —— |
| 12 | | | 36 | 39 | 33 | 29.74 | 29.87 | 92 | 84 | NE | NW | Sleet | —— | —— |
| 13 | | | 35 | 38 | 28 | 30.00 | 30.17 | 82 | 80 | NNW | NW | Cloudy | —— | —— |
| 14 | | | 34 | 40 | 34 | 29.94 | 29.85 | 82 | 82 | WSW | WNW | —— | —— | —— |
| 15 | | | 36 | 38 | 24 | 29.89 | 29.92 | 87 | 85 | NNE | NNE | Sleet | Fair | Fine |
| 16 | | | 25 | 31 | 20 | 30.02 | 29.96 | 82 | 80 | ESE | WNW | Fair | —— | —— |
| 17 | | | 22 | 30 | 19 | 29.83 | 29.87 | 85 | 83 | WSW | SW | —— | —— | Foggy |
| 18 | | | 26 | 30 | 24 | 29.94 | 29.93 | 82 | 82 | E | ENEv | Fine | —— | —— |
| 19 | | | 26 | 28 | 24 | 29.81 | 29.66 | 82 | 81 | ENE | ENE | —— | —— | Fine |

The Rain-gauge having frozen, no account was taken of the quantity of Rain fallen.

